# Minute ventilation and heart rate relationship for estimation of the ventilatory compensation point at high altitude: a pilot study

**DOI:** 10.1186/2046-7648-2-7

**Published:** 2013-03-01

**Authors:** Gabriele Valli, Mattia Internullo, Alessandro M Ferrazza, Paolo Onorati, Annalisa Cogo, Paolo Palange

**Affiliations:** 1 Lung Function Unit, Department of Public Health and Infectious Diseases, University of Rome “La Sapienza”, viale Università 37, Rome, 00185, Italy; 2 Biomedical Sport Studies Center, University of Ferrara, Via Gramicia 35, Ferrara, 44123, Italy; 3 Eleonora Lorillard Spencer Cenci Foundation, Piazzale Aldo Moro n. 5, Rome, 00185, Italy

**Keywords:** High altitude, Ventilatory compensation point, Exercise, Ventilation, Heart rate

## Abstract

**Background:**

The ventilatory compensation point (VCP) is an exercise threshold which has been used in the design of training programs in sports medicine and rehabilitation. We recently demonstrated that changes in the slope of the minute ventilation to heart rate relationship ($\Delta {\dot{V}}_{E}/\Delta \mathrm{HR}$) can be utilized for estimation of the VCP during incremental exercise at sea level (SL). We hypothesized that in hypoxic conditions, such as high altitude (HA), VCP can be also reliably estimated by $\Delta {\dot{V}}_{E}/\Delta \mathrm{HR}$.

**Methods:**

At SL and on immediate ascent to HA (5,050 m), six healthy subjects (42 ± 14 SD years) performed a maximal incremental exercise test on a cycle ergometer; O_2_ uptake (${\dot{V}}_{O_{2}}$), CO_2_ output (${\dot{V}}_{{\mathrm{CO}}_{2}}$), ${\dot{V}}_{E}$, and HR were measured breath-by-breath. The $\Delta {\dot{V}}_{E}/\Delta \mathrm{HR}$ method for VCP estimation was compared to the standard method using the ventilatory equivalent for CO_2_ (${\dot{V}}_{E}/{\dot{V}}_{{CO}_{2}}$) and end-tidal PCO_2_ (P_ET_CO_2_). The $\Delta {\dot{V}}_{E}/\Delta \mathrm{HR}$ slope values below (S_1_) and above (S_2_) VCP were computed by linear regression analysis.

**Results:**

A significant difference between S_1_ and S_2_ was observed, at SL and HA, for both the $\Delta {\dot{V}}_{E}/\Delta \mathrm{HR}$ and ${\dot{V}}_{E}/{\dot{V}}_{{\mathrm{CO}}_{2}}$ methods for VCP estimation. A good agreement between the two methods ($\Delta {\dot{V}}_{E}/\Delta \mathrm{HR}$ vs. ${\dot{V}}_{E}/{\dot{V}}_{{\mathrm{CO}}_{2}}$) was found for both environmental conditions; the mean difference ± 2 SD of ${\dot{V}}_{O_{2}}$ at VCP (VCP-${\dot{V}}_{O_{2}}$) was −22 ± 112 ml/min at SL and 39 ± 81 ml/min at HA. The VCP-${\dot{V}}_{O_{2}}$ was significantly lower at HA compared to SL; in addition, S_1_ and S_2_ mean values were significantly higher at HA compared to SL.

**Conclusion:**

At HA, VCP may be reliably estimated by the $\Delta {\dot{V}}_{E}/\Delta \mathrm{HR}$ method.

## Background

In normal individuals, minute ventilation (${\dot{V}}_{E}$) during exercise displays a more marked rapid increase when work rate exceeds the heavy-intensity domains, as a compensatory effect of the metabolic acidosis [1,2], and this threshold is called the ventilatory compensation point (VCP) [3]. The VCP has been utilized for the design of training programs in sports medicine and rehabilitation [4,5]. Practical and, in some instances, simple approaches for estimation of the VCP have been evaluated in the past by different authors. Several strategies have been proposed, based either on ${\dot{V}}_{E}$[6] or heart rate (HR) responses [7,8].

Since HR is usually reasonably linear with respect to oxygen uptake (${\dot{V}}_{O_{2}}$) over the entire work rate range [9], we have argued that the changes in the slope of increment in ${\dot{V}}_{E}$ over HR response ($\Delta {\dot{V}}_{E}/\mathrm{\Delta HR}$) can be utilized to estimate VCP during incremental exercise at sea level (SL) [10,11]. The $\Delta {\dot{V}}_{E}/\mathrm{\Delta HR}$ method has shown a good agreement with the standard method for VCP estimation (i.e., increased ventilatory equivalent for CO_2_${\dot{V}}_{E}/{\dot{V}}_{{\mathrm{CO}}_{2}}$) and decreased end-tidal PCO_2_ (P_ET_CO_2_), independently of exercise modality [11] and protocol [10]. The $\Delta {\dot{V}}_{E}/\mathrm{\Delta HR}$ method therefore may be a potentially promising method to estimate VCP during field tests or in difficult environmental conditions.

At high altitude (HA), compared to SL, ${\dot{V}}_{E}$ is greatly increased at any given work rate [12]. This may have a detrimental effect on exercise tolerance because it may lead to a significant reduction in ventilatory reserve [13]; however, reduced regional blood flow and oxygen diffusion limitation in the lungs and exercising muscles are also believed to play an important role in limiting exercise tolerance at HA [14]. The HR response at any given work rate is also increased at HA [15], although maximal HR appears to be reduced [13-16]. No data, however, are available on the slopes of the $\Delta {\dot{V}}_{E}/\mathrm{\Delta HR}$ response to exercise at HA.

In the present study, we wanted to establish the reliability of the $\Delta {\dot{V}}_{E}/\mathrm{\Delta HR}$ method for the estimation of VCP at HA, an environmental condition associated with an augmented ventilatory and cardiac response to exercise. We hypothesized that the $\Delta {\dot{V}}_{E}/\mathrm{\Delta HR}$ method at HA would be as reliable as the standard method for VCP estimation. If so, this could lead to the development of less expensive exercise equipment, i.e., without gas analyzers, that could readily be easily used in sports medicine and in high altitude studies where calibaration of gas analyzers is more difficult.

## Methods

### Subjects

Six healthy non-smoking SL resident subjects (two females, four males; Table 1) provided written informed consent to participate in the study. The procedures and protocols were approved by the National Research Council (CNR, Italy) as part of the Ev-K2-CNR research program and were conducted in accordance with the Declaration of Helsinki.

**Table 1:** Subjects' baseline characteristics

**Baseline characteristic**	**Value**
Age (years)	41 ± 15
Height (cm)	1.74 ± 0.10
BMI (kg/m^2^)	23.6 ± 2.9
FVC at SL (l, %pred)	4.70 ± 0.90, 103 ± 14
FEV_1_ at SL (l, %pred)	3.77 ± 0.75, 109 ± 16
FVC at HA (l, %pred)	4.59 ± 0.99, 100 ± 14
FEV_1_ at HA (l, %pred)	3.79 ± 0.98, 106 ± 17

FVC, forced vital capacity; FEV, forced expiratory volume; %pred, percent predicted.

### Protocols

The SL phase was conducted in Rome, Italy, at approximately 60 m of altitude. On day 1, the subjects performed standard pulmonary function tests and got familiarized with the maximal incremental exercise test. On the subsequent day, the formal maximal incremental exercise test was conducted until exhaustion.

The HA phase of the study was conducted at the CNR Pyramid Laboratory, Lobuche, Khumbu, Nepal (5,050 m, barometric pressure approximately 410 Torr). After travelling by plane through Kathmandu (1,340 m) up to Lukla (2,860 m), all subjects performed a 10-day trek to the Pyramid Laboratory with the following intermediate stops for acclimatization: 3 days at Namche Bazaar (3,450 m) and then 2 days at Pheriche (4,252 m). Symptoms of acute mountain sickness and resting arterial O_2_ saturation (SpO_2_) were monitored. After 1 day of rest at the Pyramid Laboratory, the experimental phase started. On day 2, the subjects performed standard pulmonary function tests. Calibration of the spirometer (photoelectric digital turbine, diameter 28 mm, resolution 4 ml, Micro Kit, COSMED, Rome, Italy) was performed prior to each test, using a 3-l syringe.

### Incremental exercise test

On day 3, each subject performed a maximal incremental exercise test on a cycle ergometer. The pedaling frequency was set at 60 ± 5 rpm by subjects following an electronic audio signal generated each second. The test protocol consisted (a) 2 min of rest, (b) 4 min of exercise at 20 W, (c) the incremental phase with work rate increments of 15 W/min at HA and 25 W/min at SL, (d) 6 min of recovery pedalling at 20 W. O_2_ uptake (${\dot{V}}_{O_{2}}$, standard temperature and pressure, dry (STPD)), CO_2_ output (${\dot{V}}_{{\mathrm{CO}}_{2}}$, STPD), minute ventilation (${\dot{V}}_{E}$, body temperature and pressure saturated BTPS), and end-tidal partial pressures for O_2_ and CO_2_ (P_ET_O_2_, P_ET_CO_2_) were obtained breath-by-breath and edited to exclude occasional outlying breaths (>±4 SD of the local mean) as a result of coughs, swallows, sighing, or gasping. A 10-s average was utilized for subsequent analysis.

For VCP estimation by analysis of the $\Delta {\dot{V}}_{E}/\mathrm{\Delta HR}$ relationship (${\mathrm{VCP}}_{{\dot{V}}_{E}/\mathrm{HR}}$), we utilized a least squares regression analysis. Using a dedicated software, we applied a ‘best fit’ line to the data which extended from end-exercise back to the sub-maximal point at which the linearity was lost (S_2_ region); this breakpoint was compared with the standard approach for VCP estimation (${\mathrm{VCP}}_{{\dot{V}}_{E}/{\dot{V}}_{{\mathrm{CO}}_{2}}}$) based on the ${\dot{V}}_{E}/{\dot{V}}_{{\mathrm{CO}}_{2}}$ relationship, where ${\dot{V}}_{E}$ started to change out of proportion of ${\dot{V}}_{{\mathrm{CO}}_{2}}$ and P_ET_CO_2_ started to fall [17]. From the aforementioned breakpoint, a second best fit line (S_1_) was applied to the data extending from the end of the warm-up phase upwards into the exercise data. If a second breakpoint was discernible, the S_1_ line was then divided in two different regions (S_1_” from the breakpoint to ${\mathrm{VCP}}_{{\dot{V}}_{E}/\mathrm{HR}}$ and S_1_’ from the end of warm-up up to the observed breakpoint), and the HR value at this breakpoint was then compared with the value observed at lactate threshold (θ_L_, estimated by the ‘V-slope method’ [3] and supported by standard ventilatory equivalent and end-tidal gas tension criteria [17]).

### Equipment

The incremental exercise test was performed on a mechanically braked cycle ergometer (828E, Monark Exercise AB, Varberg, Sweden). Prior to each test, the zero-load setting on the ergometer was checked, and a calibration was performed with a 4-kg weight. Ventilatory and pulmonary gas exchange variables were measured breath-by-breath in all tests using a portable system (K4b^2^, COSMED) which has been previously validated at HA [18]; the accuracy of the telemetric system has been previously established [18,19]. The system comprised a face mask, analyzer unit (containing O_2_ and CO_2_ gas analyzers), heart rate monitor, and battery. The analyzer unit with battery pack, face mask, and tubing (weight 0.8 kg) was attached to the subject with a harness and connected to a personal computer by an Ethernet cable connection. The face mask contained a turbine for measurement of volume and flow; calibration was performed with a 3-l syringe (Hans Rudolph, Kansas City, MO, USA) over a range of different flow profiles. Respired gas, sampled continuously from a port within the turbine via a Nafion polymer capillary (PermaPure©, Toms River, NJ, USA), was analyzed at 100 Hz using rapid-response O_2_ (polarographic) and CO_2_ (infrared) analyzers (mean response time 120 ms) which were automatically thermostated and compensated for ambient variations in barometric pressure, humidity, and environmental temperature. Analyzers' calibration was performed using two precision-analyzed gas mixtures spanning the respired range. The volume and gas concentration signals were sampled and digitized every 10 ms, and time-aligned, i.e., correcting for the transport delay between the turbine and gas analyzers and for the analyzer rise time [20]. HR was measured from a chest strip and recorded every breath. SpO_2_ was monitored non-invasively by finger pulse oximetry (Masimo Rad-5, Masimo Corporation, Irvine, CA, USA).

### Statistical analyses

Differences among measured responses were determined by a Student's paired t test. Pearson's product–moment correlation coefficient (R^2^) was used to identify correlations between criterion variables. The level of statistical significance was set at P < 0.05. Group data are presented as mean ± SD. The limits of agreement between the ${\dot{V}}_{E}/{\dot{V}}_{{\mathrm{CO}}_{2}}$ and the $\Delta {\dot{V}}_{E}/\mathrm{\Delta HR}$ methods for VCP estimation were evaluated by the Bland-Altman analysis [21], where the individual differences are plotted against their respective means. We proceeded with such a type of analysis if a significant linear correlation between methods was previously observed. The same statistical approach was also performed to compare the correspondence between θ_L_ and the breakpoint between S_1_” and S_1_’ in the $\Delta {\dot{V}}_{E}/\mathrm{\Delta HR}$ relationship, when the latter was detectable.

## Results

The main results of the incremental tests are illustrated in Table 2. At HA, compared to SL, a significant reduction in ${\dot{V}}_{O_{2}\mathrm{peaks}}$, WR_peak_, HR_peak_, SpO_2peak_, and θ_L_ was observed; on the contrary, ${\dot{V}}_{\mathrm{Epeak}}$ at HA was appreciably higher.

**Table 2:** Results of the incremental exercise tests at HA and SL

	**HA**	**SL**	***P *value**
SpO_2peak_ (%)	72.8 ± 7.3	98 ± 1.4	<0.001
WR_peak_ (W)	138 ± 26	208 ± 44	<0.01
${\dot{V}}_{O_{2\mathrm{peak}}}$ (ml/min)	1,690 ±533	2,227 ± 511	<0.01
${\dot{V}}_{{\mathrm{CO}}_{2\mathrm{peak}}}$ (ml/min)	2,060 ± 570	2,820 ± 780	<0.01
${\dot{V}}_{\mathrm{Epeak}}$ (l/min)	123.4 ± 33.3	91.3 ± 28.6	<0.01
*θ*_L_ (ml/min)	1,015 ± 222	1,302 ± 241	<0.01
HR_peak_ (bpm/min)	147 ± 13	168 ± 12	<0.001

**Figure 1:**
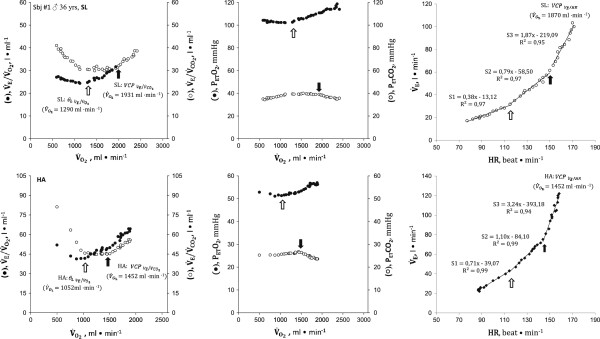
**Representative subject #6 (male, 56 years).** Exercise response variables in a representative subject at SL (*empty circle*) and at HA (*filled circle*). The *black arrow* indicates VCP; the *white arrow* indicates the *θ*_L_. A single breakpoint in the ${\dot{V}}_{E}/\mathrm{HR}$ at VCP was clearly identifiable, both at SL and at HA (see text for further comments).

**Figure 2:**
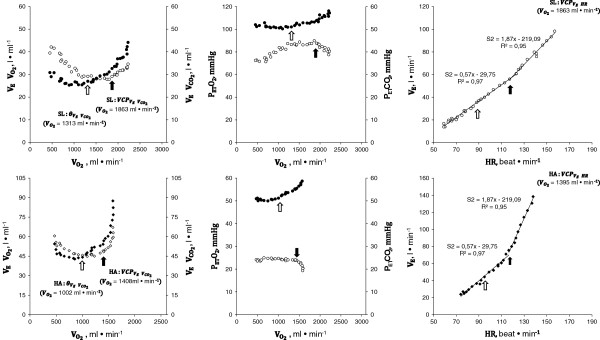
**Representative subject #1 (male, 36 years).** Exercise response variables in a representative subject at SL (*empty circle*) and at HA (*filled circle*). The *black arrow* indicates VCP; the *white arrow* indicates the *θ*_L_. In this subject, a second and less evident breakpoint ${\dot{V}}_{E}/\mathrm{HR}$ that occurred at the HR value observed at *θ*_L_ was identifiable (see text for further comments).

**Figure 3:**
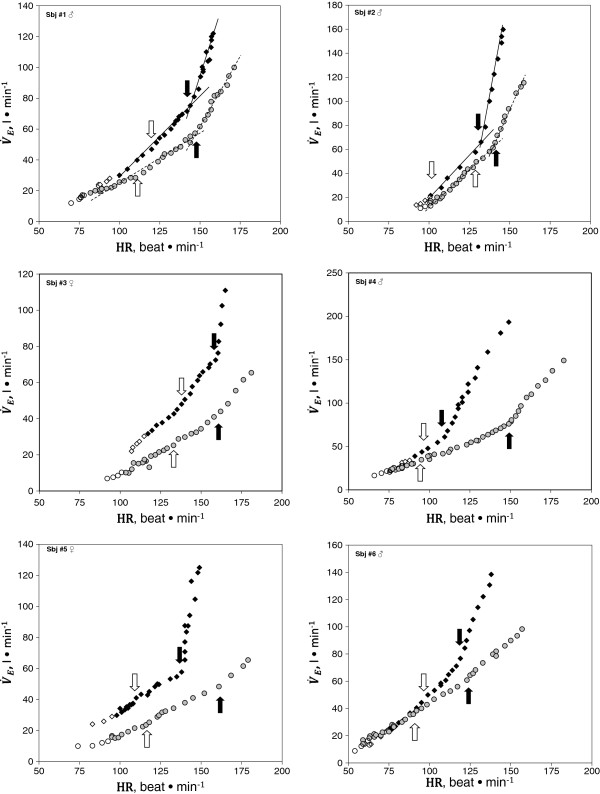
**Exercise **${\dot{V}}_{E}/\mathrm{HR}$**relationship in all subjects.** Exercise ${\dot{V}}_{E}/\mathrm{HR}$ response in the six subjects studied at SL (*circle*) and at HA (*square*)at free will (*empty symbol*) and with effort (*filled symbol*). The *black arrow* indicates VCP; the *white arrow* indicates the *θ*_L_ (see text for further comments).

Figures 1 and 2 show $\Delta {\dot{V}}_{E}/\Delta_{O_{2}}$, ${\dot{V}}_{E}/{\dot{V}}_{{\mathrm{CO}}_{2}}$, P_ET_O_2_, P_ET_CO_2_ vs. ${\dot{V}}_{{\mathrm{CO}}_{2}}$, and $\Delta {\dot{V}}_{E}/\mathrm{\Delta HR}$ relationship in two representative subjects at SL (upper panels) and HA (lower panels). As shown in Figure 3, in all subjects, a breakpoint in $\Delta {\dot{V}}_{E}/\mathrm{\Delta HR}$, which occurred at the VCP estimated by the ${\dot{V}}_{E}/{\dot{V}}_{{\mathrm{CO}}_{2}}$ method, was clearly discernible both at SL and HA. No significant differences were found in ${\dot{V}}_{O_{2}}$ measured at VCP (${\dot{V}}_{{\mathrm{CO}}_{2}}$ -VCP; Table 3) between methods utilized to identify the threshold (VCP-$\Delta {\dot{V}}_{E}/\mathrm{\Delta HR}$ vs. VCP-${\dot{V}}_{E}/{\dot{V}}_{{\mathrm{CO}}_{2}}$).

**Table 3:** **VCP, S**_**1**_**, and S**_**2 **_**values at HA and SL**

	**HA**	**SL**	***P *value**
$\mathrm{VCP}-\Delta {\dot{V}}_{E}/\Delta \mathrm{HR}$ (ml/min)	1,325.3 ± 214.7	1,822.0 ± 371.6	<0.05
$\mathrm{VCP}-{\dot{V}}_{E}/{\dot{V}}_{{\mathrm{CO}}_{2}}$ (ml/min)	1,315.1 ± 180.8	1,839.1 ± 359.5	<0.05
$S_{1_{{\dot{V}}_{E}/\mathrm{HR}}}$ (l/bp)	1.09 ± 0.33	0.76 ± 0.32	<0.01
$S_{2_{{\dot{V}}_{E}/\mathrm{HR}}}$ (l/bp)	4.8 ± 2.5	1.4 ± 0.6	<0.05
$S_{1_{{\dot{V}}_{E}/{\dot{V}}_{{\mathrm{CO}}_{2}}}}$	0.043 ± 0.005	0.025 ± 0.001	<0.01
$S_{2_{{\dot{V}}_{E}/{\dot{V}}_{{\mathrm{CO}}_{2}}}}$	0.114 ± 0.052	0.047 ± 0.01	<0.05
$S_{2}/S_{1_{{\dot{V}}_{E}/\mathrm{HR}}}$	4.5 ± 1.9	2.0 ± 0.8	NS
$S_{2}/S_{1_{{\dot{V}}_{E}/\dot{V}{\mathrm{CO}}_{2}}}$	2.7 ± 1.5	1.9 ± 0.4	NS

NS, not significant.

**Figure 4:**
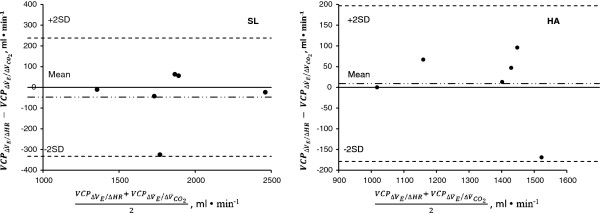
**Bland-Altman analysis.** The Bland-Altman analysis confirmed the agreement between the ${\dot{V}}_{E}/{\dot{V}}_{{\mathrm{CO}}_{2}}$ and ${\dot{V}}_{E}/\mathrm{HR}$ methods in estimating VCP, both at SL and at HA.

The Bland-Altman analysis confirmed the agreement between the ${\dot{V}}_{E}/{\dot{V}}_{{\mathrm{CO}}_{2}}$ and $\Delta {\dot{V}}_{E}/\mathrm{\Delta HR}$ methods in estimating VCP, both at SL and at HA (Figure 4, Table 3); the mean bias ± 95% confidence interval of the between-method differences were −22 ± 112 ml/min of ${\dot{V}}_{O_{2}}$ for the SL protocol and 39 ± 81 ml/min of ${\dot{V}}_{O_{2}}$ for the HA protocol (Figure 4).

Although in some instances a lower breakpoint in the S_1_ region was observed (four out of six subjects at SL and one out of six at HA), no significant agreement between such a breakpoint and the θ_L_ was obtained in terms of HR and ${\dot{V}}_{O_{2}}$ values. $\;{\dot{V}}_{{\mathrm{Otry11}}_{2}}$-VCP was significantly lower at HA (approximately 500 ml of ${\dot{V}}_{O_{2}}$ less) independently of the method utilized to estimate it, as shown in Table 2.

S_1_ and S_2_ at HA were significantly higher for both $\Delta {\dot{V}}_{E}/\mathrm{\Delta HR}$ and ${\dot{V}}_{E}/{\dot{V}}_{{\mathrm{CO}}_{2}}$ methods (Table 3). The ratio S_2_/S_1_ tended to be higher at HA regardless of the estimation method utilized (Table 3), and this was particularly so for the $\Delta {\dot{V}}_{E}/\mathrm{\Delta HR}$ method.

## Discussion and conclusions

The main findings of this study are as follows: (1) VCP is clearly identifiable during incremental exercise at HA; (2) in conditions of persistent hypoxia, such as that elicited by HA exposure, VCP can be easily estimated by the $\Delta {\dot{V}}_{E}/\mathrm{\Delta HR}$ analysis. In the present study on young healthy individuals, applying standard methods for the identification, VCP could be identified both at SL and HA. Our data are in accordance with the observation of Agostoni et al., who demonstrated that at HA the VCP was clearly identifiable [22].

Of note, we observed a 30% reduction in ${\dot{V}}_{O_{2}}$-VCP when moving from SL to HA. The lower VCP at HA likely reflected the shortening of the isocapnic buffering period because of the increased hypoxic sensitivity of the carotid chemoreflex [22]. A further influence was presumably the premature development of metabolic acidosis during exercise at HA, as demonstrated by the lower θ_L_ likely due both to the reduction in arterial O_2_ content and impaired convective O_2_ transport to the exercising muscles [23].

Importantly, as demonstrated by the Bland-Altman analysis, we were able to demonstrate that the $\Delta {\dot{V}}_{E}/\mathrm{\Delta HR}$ approach is a reliable method for VCP estimation during incremental exercise at HA. The limits of agreement and the coefficient of variation between the ${\dot{V}}_{E}/{\dot{V}}_{{\mathrm{CO}}_{2}}$ and $\Delta {\dot{V}}_{E}/\mathrm{\Delta HR}$ methods for VCP estimation are quite narrow and likely to fall within the between-day intra-subject ${\dot{V}}_{O_{2}}$ variability [24].

The results of the present study are in agreement with a previous work of our group that demonstrated the reliability of the $\Delta {\dot{V}}_{E}/\mathrm{\Delta HR}$ breakpoint for VCP estimation at SL during incremental exercise [10,11]. At SL, assuming a linear relationship between ${\dot{V}}_{E}$ and HR (at least up to the heavy-intensity domain), the steepening of the $\Delta {\dot{V}}_{E}/\mathrm{\Delta HR}$ slope is more evident above VCP where (1) a steeper increase in ${\dot{V}}_{E}/O_{2}$ relationship is expected [9] and (2) the HR response could either maintain the same linearity as for more moderate exercise or in some instances (depending on exercise performance and protocol) reduce its rate of increment with respect to work rate [25]. Interestingly, at HA, we observed higher S_1_ and S_2_$\Delta {\dot{V}}_{E}/\mathrm{\Delta HR}$ slopes. These findings are in accordance with previous studies demonstrating that ${\dot{V}}_{E}$ at rest and at any given work rate during exercise is increased at HA because of the greater hypoxic drive [12-26] and also because of the reduction in the rate of increase in HR between rest and peak exercise that is commonly observed at HA [15,16]. Moreover, compared to SL, we observed an increase in the S_2_/S_1_ ratio for $\Delta {\dot{V}}_{E}/\mathrm{\Delta HR}$ at HA. This finding suggests that S_2_ is influenced both by hypoxic ventilatory drive and possibly by an augmented contribution to ${\dot{V}}_{E}$ from the metabolic acidosis above the VCP [1]. However, our reasoning remains speculative as, for technical reasons, we were unable to measure arterial (or arterialized) [lactate] or pH during the exercise.

Below VCP, an earlier (in the S_1_ region) but less evident breakpoint in $\Delta {\dot{V}}_{E}/\mathrm{\Delta HR}$ was discernible in some instances (5 out of 12), but only in one out five cases such a breakpoint was coincident with the anaerobic threshold.

We are aware that our study has limitations, particularly with regard to the small sample size and the constrained characteristics of our study population, which prevented the evaluation of factors such as age, sex, and level of fitness, each of which is known to influence the variables of interest. Also, we are aware (1) that the choice of a rapid work rate incremental protocol may have influenced the results utilized, i.e., different results may be observed if a slower work rate increment protocol is used, and (2) of the lack of validation for pulmonary gas exchange measurement at HA. We took precautions to limit errors in the calibration procedures, with all equipment being calibrated before each test and all tests being performed indoors in the Pyramid Laboratory at an ambient temperature of approximately 20°C and the necessary corrections for barometric pressure, humidity, and environmental temperature being applied through the dedicated software. Thus, our investigation should be considered as a pilot study conducted at extreme altitude, with the conclusions only being applicable to healthy untrained young adults.

In conclusion, we were able to demonstrate the reliability of the $\Delta {\dot{V}}_{E}/\mathrm{\Delta HR}$ method for the estimation of VCP at HA in a small group of sea-level residents, an environmental condition associated with different ventilatory and cardiac responses to exercise compared to SL. The attraction of the $\Delta {\dot{V}}_{E}/\mathrm{\Delta HR}$ method is that it is a less expensive method for VCP estimation, compared to those that utilize expired gas measurements, and may therefore be utilized in sports medicine and in extreme conditions such as high altitude.

## Competing interests

The authors declare that they have no competing interests.

## Authors’ contributions

GV was the principal investigator, conceived the study, participated in all phases of the research, and drafted the manuscript. MI and AMF participated in the design of the study and in the data collection and performed the statistical analysis. PO helped conceive the study, participated in its design and coordination, and helped draft the manuscript. AC participated in the logistic organization, coordinated the research during the HA phase of the study that was conducted at the CNR Pyramid Laboratory, Lobuche, Khumbu, Nepal, and critically revised the final draft. PP helped conceive the study, supervised all phases of the research, helped in its design and coordination, and revised critically the manuscript. All authors read and approved the final manuscript.
